# Supplementary figures and images for: Evolutionary Implications of the microRNA- and piRNA Complement of Lepidodermella squamata (Gastrotricha)
Source: Noncoding RNA. 2019 Feb 22;5(1):19. doi: 10.3390/ncrna5010019 (PMC6468455; doi:10.3390/ncrna5010019)

LINE/RTE-X  
LINE  
LTR/Copia

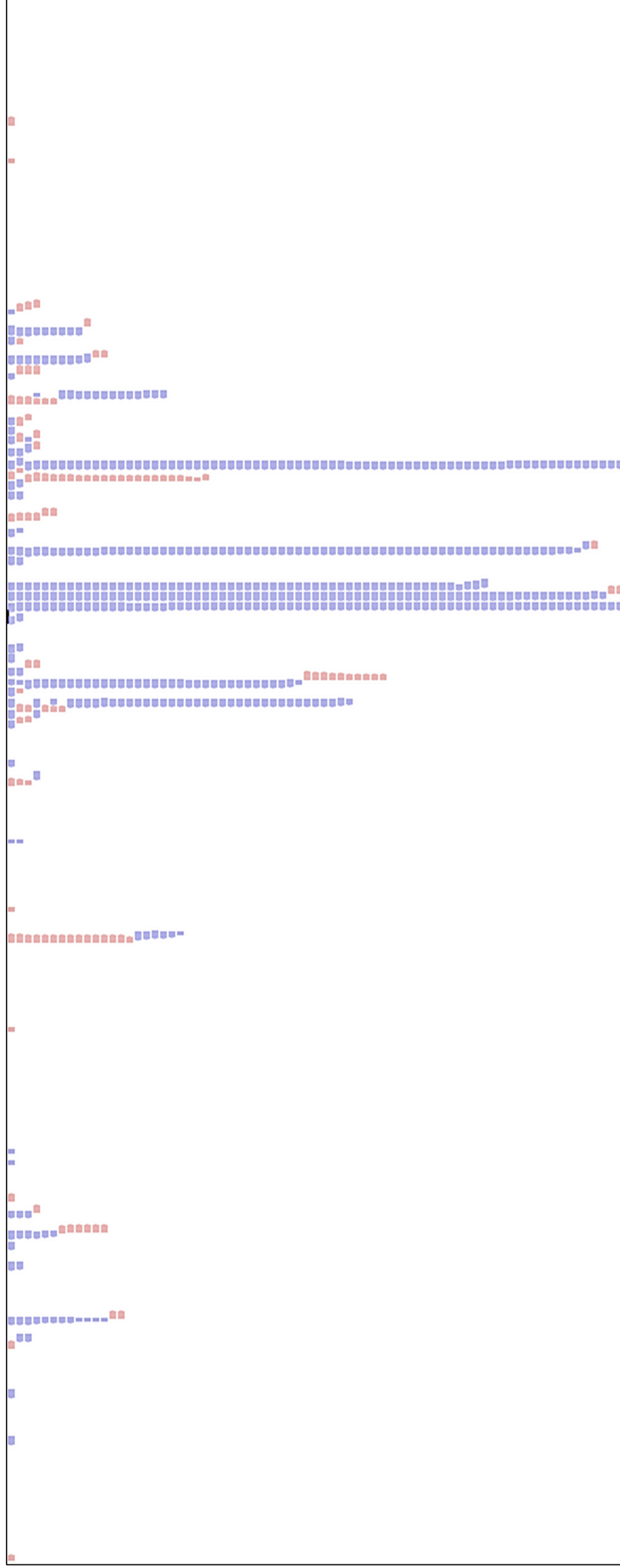

piRNA cluster #4: scaffold386\_size93225:37,926-42,144

Supplement: Supplementary file 1 [file ncrna-05-00019-s001.zip › Supplementary-FigureS4.pdf]

a

# PAZ domain

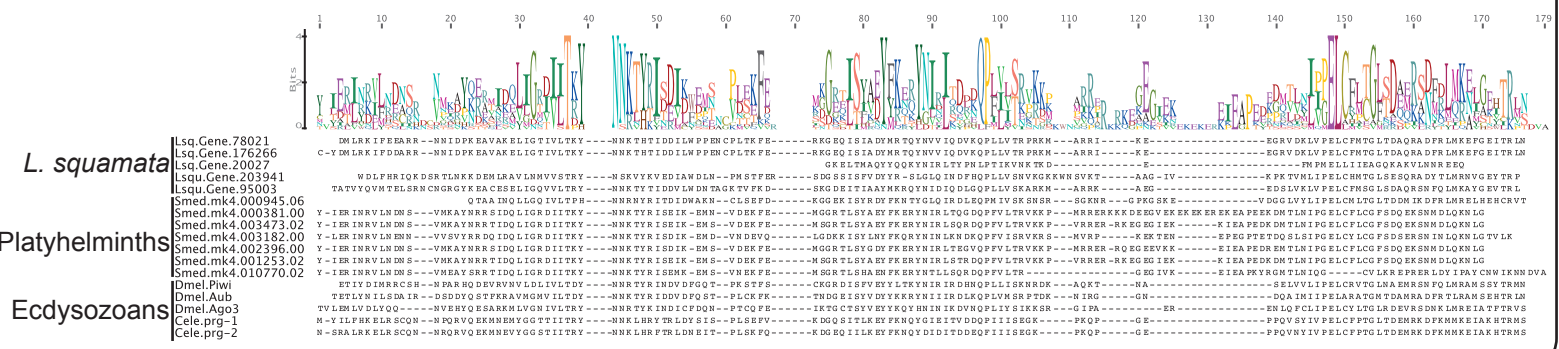

b

# Piwi domain

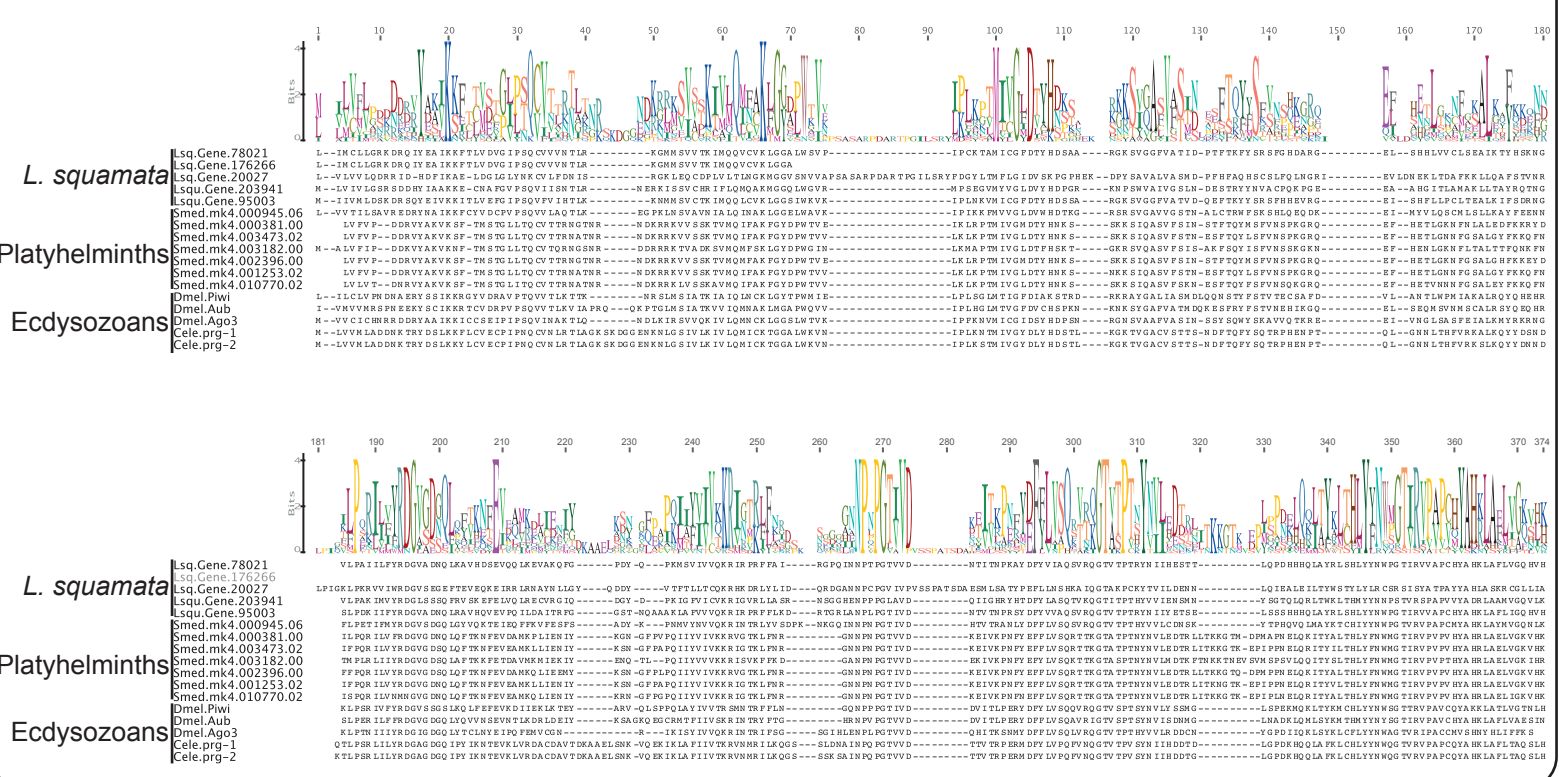

Supplement: Supplementary file 1 [file ncrna-05-00019-s001.zip › Supplementary-FigureS5.pdf]

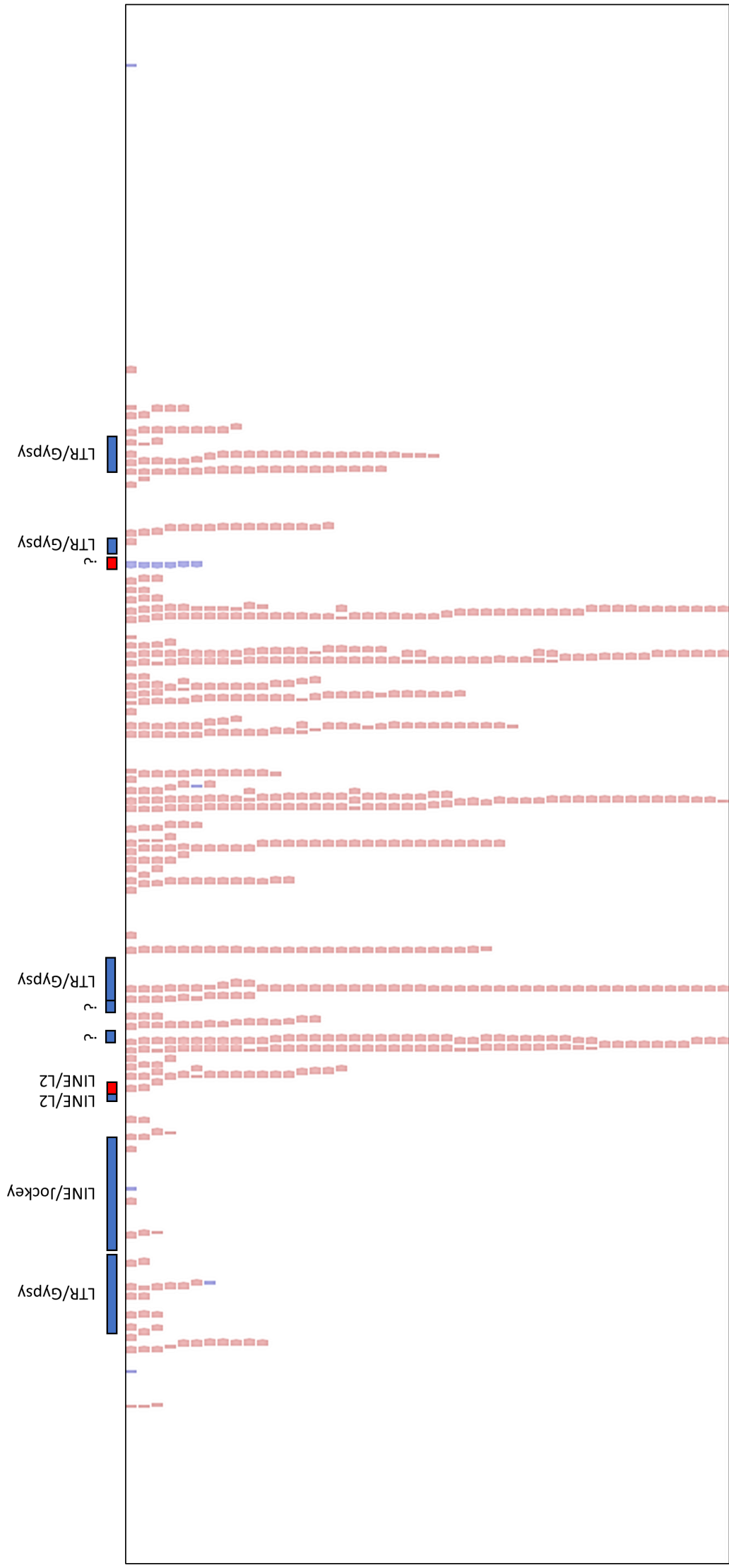

piRNA cluster #1: scaffold95\_size236010:16,013-20,557

Supplement: Supplementary file 1 [file ncrna-05-00019-s001.zip › Supplementary-FigureS1.pdf]

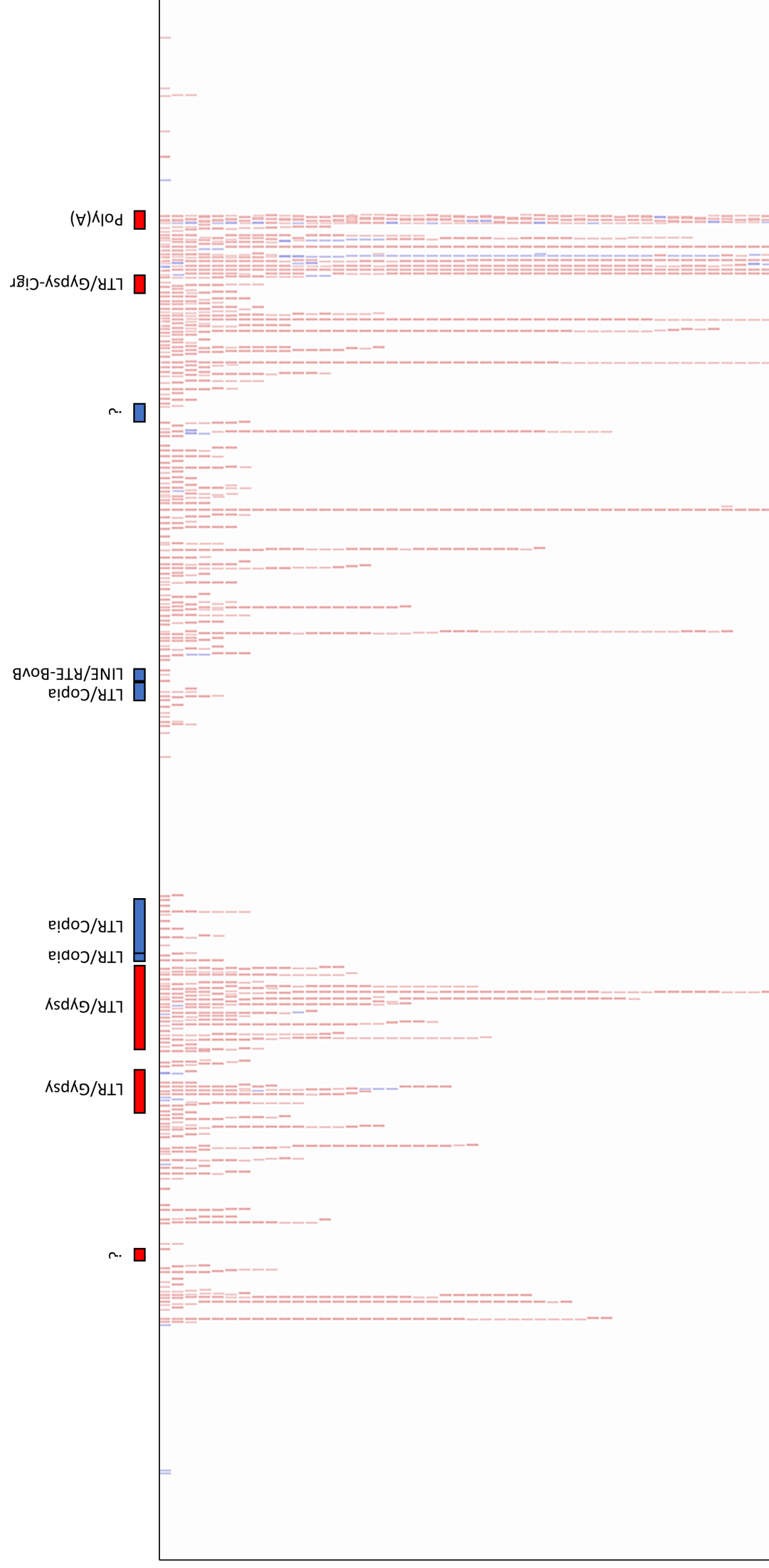

piRNA cluster #2: scaffold171\_size172051:144,160-158,000

Supplement: Supplementary file 1 [file ncrna-05-00019-s001.zip › Supplementary-FigureS2.pdf]

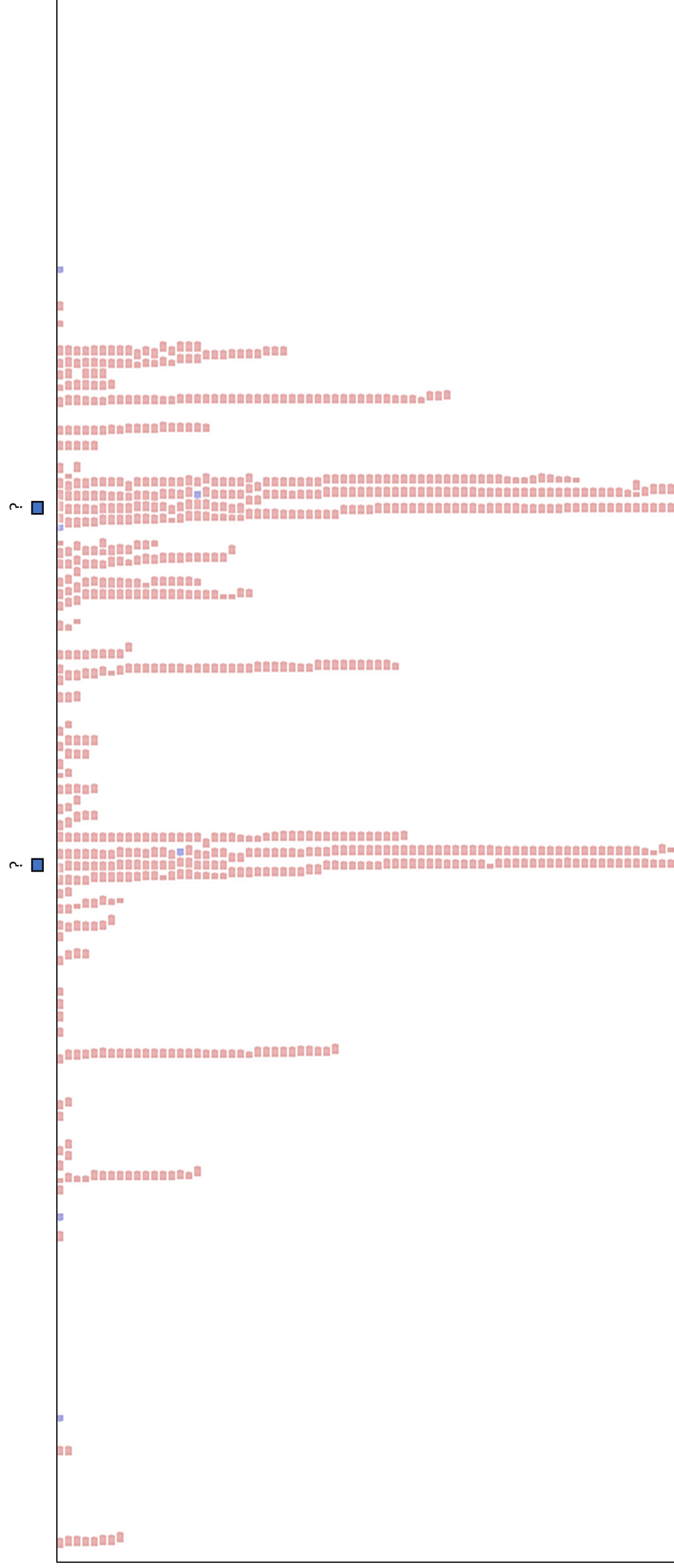

Supplement: Supplementary file 1 [file ncrna-05-00019-s001.zip › Supplementary-FigureS3.pdf]
